# Evaluation of Trauma Patient Transport via Emergency Medical Services in Tabriz

**DOI:** 10.5812/traumamon.4634

**Published:** 2012-05-26

**Authors:** Alireza Ala, Samad Shams Vahdati, Giti Seyyedghiasi

**Affiliations:** 1 Department of Emergency Medicine, Tabriz University of Medical Sciences, Tabriz, IR Iran; 2 Tabriz University of Medical Sciences, Tabriz, IR Iran

**Keywords:** Wound and Injuries, Emergency Medical Services

## Dear Editor,

Emergency Medical Services (EMS) providers have the responsibility of offering care and transporting traumatic patients to hospitals. EMS is a crucial part of the healthcare system and the public’s emergency medical safety net (1). There is little research addressing the effectiveness of EMS practice. Considering this, we decided to study the condition of trauma patients transported to the Imam Reza Hospital by Emergency Medical Services in Tabriz from March to October 2010.

In one research done at Saint Mary’s Hospital by Myers et al, (2009), 20% (9.42) of patients who had spinal fractures and had indications for immobilization, transfer was not performed by EMS (2). In another research done in USA by Peery et al.(2007) suggested that many patients were not well immobilized on arrival at the Emergency Department (3). In a clinical review by Coates (2002) from the authors’ personal experience of working in prehospital care at the London Helicopter Emergency Medical Service, showed that providing excellent medical treatment at a road crash requires special training and experience (4).

An Iranian study by Khorasani et al., (2009) indicated that involvement of laypersons could be a key factor in making post-crash management more effective and that system improvements were crucial (5).

In our study, 200 trauma patients were admitted to our hospital during the study period. Out of which, 150 (75%) were male and 50 (25%) were female. Mean age was 39 ± 16.08 years and age of our patients ranged from 18 to 88 years, which means about 20% of them were 25 years-old. Automobile accidents were the most frequent (37.5%) cause of injury. We found that the highest mean age of trauma patients was in automobile-passenger accidents and the lowest was in motorcycle accidents (P < 0.001); 125 (62.5%) of trauma patients had intravenous (IV) line access of which 70 (35%) of them received fluid therapy. The EMS personnel immobilized cervical spine in 17.5% of patients. EMS providers placed back board only for 10 (5%) patients of while 55 of them needed it; 75 patient had external bleeding which was controlled in 6.6% of patients; 40 patients needed limb immobilization but it was only done for 9 patients and which was correct in 5 patients. The personnel performed airway management in 5% of patients.

In general the EMS providers failed to perform spinal immobilization and control external bleeding in trauma patients. Pre-hospital personnel did not follow any valid guidelines and there was no relation between indications and measures taken by EMS personnel.
